# Feasibility of heart girth models in estimating live weight of fat‐long‐tailed sheep

**DOI:** 10.1002/vms3.476

**Published:** 2021-03-22

**Authors:** Jane Wamatu, Ashraf Alkhtib

**Affiliations:** ^1^ International Center for Agricultural Research in Dry Areas (ICARDA) Addis Ababa Ethiopia; ^2^ School of Animal, Rural and Environmental Sciences Nottingham Trent University Nottingham UK

**Keywords:** heart girth, linear, nonlinear, prediction error, regression

## Abstract

Fat deposition in the brisket of Ethiopian fat‐long‐tailed sheep may interfere with the correlation between heart girth (HG) and live weight (LW), bringing into question the accuracy of HG models for LW prediction that are currently in use. This study assessed the accuracy of published HG‐based prediction models of the live weight of Ethiopian sheep breeds. Furthermore, the study identified accurate and robust models that predict the LW of the sheep using HG. Live weight and HG of 1,020 sheep from Bonga, Adilo and Horro breeds were measured. First, data collected from the study was used to gauge the preciseness of previously published prediction models of each breed. Second, the data of individual breeds were divided into a calibration set for model construction and a validation set for model validation. Live weight was regressed on HG to construct simple linear, Box‐Cox, quadratic and allometric prediction models. Prediction error of published models was >20%. Models constructed for each breed did not differ in R^2^. However, only simple linear models with transformed LW (Adilo: Log_10_(LW) = 0.408 + 0.015*HG, Bonga: Log_10_(LW) = −36.6 + 0.882*HG, Horro: LW^0.5^ = −1.26 + 0.085*HG) had homogenous residuals and prediction error of ≤ 10%. Heart girth models currently used to predict LW of Adilo, Bonga and Horro sheep of Ethiopia are not sufficiently accurate as they have PE higher than 10%. Prediction models generated by the current study could replace the published models for an accurate estimation of LW of the three breeds for husbandry, marketing and veterinary purposes.

## Abstract 2

The published models currently in use are inaccurate in estimating live weight of fat‐long‐tailed Ethiopian sheep The study generated simple and feasible heart girth models to predict live weight of fat‐long‐tailed Ethiopian sheep.

## 1 INTRODUCTION

Live weight (LW) and LW change of sheep are important for aspects of nutrition, management, breeding and husbandry. They are vital in determining growth, feed conversion efficiency (Veerkamp, 1998), readiness for marketing or slaughtering (Sawyer et al., 1991) and adequate veterinary medications (Machila et al., 2008). Conventional weighing scales are the key standard to determine the LW of sheep provided they are well‐calibrated, however, in rural areas, weighing scales are rarely used by farmers because they are expensive as well as labour and time intensive. Scale calibration and maintenance require skilled technicians who are rarely found in rural areas. Farmers, therefore, rely on estimating weights of livestock without recourse to validated weighing methods. Eye‐balling as an alternative for direct weighing of sheep to estimate LW has been demonstrated to lack accuracy and is prone to error (Machila et al., 2008). Heart girth (HG) has been repeatedly demonstrated to be a useful and robust proxy for the use of scales in LW estimation of sheep (Atta et al., 2004; Sowande & Sobola, 2008). Predictive models of LW based on HG have been reported for a mixture of Ethiopian highlands sheep as well as for individual breeds (Table 1).

**TABLE 1:** Summary of studies investigating the relationship between heart girth and live weight for Ethiopian sheep

Reference	Sheep description			Model			
	Breed	*n*	Live weight (kg)	Sex	*R* ^2^	Constant	Slope
Taye et al. (2016)	Adilo	512	16–50	Mixed	0.804	−39.4	0.968
Edea et al. (2008)	Bonga	688	31.9 ± 0.19	Female	0.62	−33.34	0.9
Edea et al. (2008)				Male	0.77	−40.95	0.99
Edea et al. (2008)	Horro	816	27.7 ± 0.21	Female	0.54	−36.13	0.86
			31.7 ± 1.23	Male	0.81	−39.96	1.03

Note

*R* ^2^: coefficient of determination.

Sheep, with a population of 30 million head (FAOSTAT, 2017), are the key component of the livestock farming system in Ethiopia. They are an important source of income, meat, skin, wool and manure (Gizaw et al., 2012). Ethiopian sheep are categorized into four groups (sub‐alpine short‐fat‐tailed, highland long‐fat‐tailed, lowland fat‐rumped and lowland thin‐tailed) based on their ecological distribution and tail types (Gizaw et al., 2012). Highland long‐fat‐tailed types are the best source of mutton and are well adapted to wet highlands. Adilo (also referred to as Doyogena), Bonga and Horro breeds have high growth potential highland long‐fat‐tailed sheep of Ethiopia (Gizaw et al., 2012).

Adilo, Bonga and Horro sheep are large, long and fat‐tailed hair types with deposits of fat below the lower jaw and in the brisket (Galal, 1983). The fat deposition might weaken the correlation between LW and HG, by protecting HG from changing due to the change in LW, leading to limited predictability of the prediction models. Existing predictive models for Ethiopian sheep (Table 1) were examined and it was determined that they were generated by simply regressing of LW on HG, then models were recommended with maximum R^2^ without reporting on the prediction error. Therefore, these models might result in errors higher than the accepted margins of 20% and 10% for veterinary and production purposes, respectively (Goopy et al., 2017) when predicting LW of Adilo, Bonga and Horro sheep. Another key criticism of these models is that they were not validated against independent sheep data.

Therefore, this study aimed to develop simple and robust predictive models to estimate LW in Adilo, Bonga and Horro sheep. Furthermore, the accuracy of models developed previously for these breeds and other indigenous sheep was evaluated.

## 2 MATERIALS AND METHODS

This study has been approved by the ethical committee of the International Centre of Agricultural Research in Dry Areas.

### 2.1 Study areas and sheep population

Three hundred and twenty heads of Adilo (56 males and 254 females), 321 (76 males and 234 females) heads of Bonga and 409 (61 males and 339 females) heads of Horro sheep were drawn from Areka ARC located in Southern Nations Nationalities and People's Region (7°4′N 37°42′E; 1,774 m a.s.l.), Jimma Agricultural Research Center, located in Oromia region (7°46′N 36°00′E; 1,753 m a.s.l.) and Bako Agricultural Research Center located in Oromia region (9°34′N 37°06′E; 1,659 m a.s.l.). The age of sheep ranged from 4 to 144 months in Adilo, 6 to 86 months in Bonga and 4 to 96 months in Horro. All sheep in this study were kept on natural pasture and supplemented with straws (barley, wheat, tef) among other locally available resources in line with respective farmer practice. Sick and/or pregnant sheep were excluded (10, 11 and 9 pregnant sheep for Adilo, Bonga and Horro, respectively).

### 2.2 Measurements for model development

Simultaneous measurements of LW and HG for all sheep were undertaken at their respective sites after overnight fasting. Live weight was measured gravimetrically using a portable spring‐dial hoist scale (Camry, NTB, Camty company), with a capacity of 100 kg and precision of 50 g. The scale was calibrated using standard weights, after which 10 sheep were weighed in three replications to confirm the reliability of LW measurements. Scales were further calibrated at 50‐sheep measurement intervals. Heart girth was measured as the body circumference immediately behind the front shoulder at the fourth ribs, posterior to the front leg using an ordinary tape held with 1kg tension using a light spring balance. Three trained persons were mainly collecting the data. One person was holding sheep and another person was taking the measurement. A third person was recording measurement data. Data were collected in 3 days (one day for each breed).

### 2.3 Data analysis

Outliers in the data were screened using the interquartile range method (Zwillinger & Kokoska, 2003). Since the accuracy of weighing scales may decline with successive LW measurements of sheep, the relationship between LW and the serial number of sheep (order of sheep during the measurement process) was visually presented to depict the distribution of LW across the measurement process.

The probability distribution of LW and HG was identified using normal Q‐Q plots. Thereafter, the data of the study was divided into two sets, a calibration set and a validation set for each breed using the Puchwein algorithm (Puchwein, 1988).

Linear, quadratic and allometric models, best describe the relationship between LW and HG in ruminants (Goopy et al., 2017; Lesosky et al., 2013). Therefore, a simple linear regression model, a simple linear model with Box‐Cox transformed LW, a quadratic model and an allometric model (Table 2) were constructed. Optimum power of transformation of LW was identified using a likelihood maximized Box‐Cox transformation (Box & Cox, 1964) with boundaries of −3 and +3 and a step of 0.1 Log‐likelihood of *λ* was used to identify the best power of transformation.

**TABLE 2:** Performance of the models for estimating live weight of Adilo, Bonga and Horro sheep using heart girth

Breed	Model	Performance parameters						
		*R* ^2^	RSR	MB	SB	CCC	CE
Adilo	Linear: LW = a + b*HG	0.79	0.464	<0.001	<0.001	0.881	20.9
	Box cox: Log_10_(LW) = a + b*HG	0.82	0.425	<0.001	<0.001	0.749	6.13
	Quadratic: LW = a + b*HG + c*HG^(2)^	0.8	0.451	<0.001	<0.001	0.886	20.8
	Allometric: a*HG^(b)^	0.796	0.45	<0.001	<0.001	0.886	20.8
Bonga	Linea: LW = a + b*HG	0.58	0.651	<0.001	<0.001	0.732	23.8
	Box cox: Log_10_(LW) = a + b*HG	0.59	0.632	<0.001	<0.001	0.723	7.09
	Quadratic: LW = a + b*HG + c*HG^(2)^	0.58	0.661	<0.001	<0.001	0.734	24.2
	Allometric: a*HG^(b)^	0.58	0.647	<0.001	<0.001	0.734	24.1
Horro	Linear: LW = a + bHG	0.8	0.444	<0.001	<0.001	0.879	21.6
	Box cox: LW^0.5^ = a + b*HG	0.81	0.435	<0.001	<0.001	0.881	10
	Quadratic: LW = a + b*HG + c*HG^(2)^	0.811	0.439	<0.001	<0.001	0.857	20.6
	Allometric: a*HG^(b)^	0.811	0.438	<0.001	<0.001	0.857	20.7

Abbreviations: CCC, the concordance correlation coefficient; CE, 95th percentile of calibration error; HG, heart girth (cm); LW, live weight (kg); MB: mean bias; *R* ^2^: coefficient of determination; RSR, the root mean square prediction error to standard deviation ratio; SB, slope bias; *p* was <0.001 for all constructed models.

Performance of the constructed models in predicting LW was evaluated using the coefficient of determination (*R* ^2^), the root mean square prediction error to standard deviation ratio (RSR), mean bias (MB), slope bias (SB), concordance correlation coefficient (CCC) and calibration error (CE). The RSR was calculated as follows:

$$\text{RSR}=\frac{\sqrt{\frac{\sum_{i=1}^{n}{\left(P_{i}‐O_{i}\right)}^{2}}{n}}}{S_{O}}$$

where O*_i_* is the observed value, P*_i_* is the predicted value and S_O_ is the standard deviation of the observed values. The lower the RSR value, the better the predictability (Moriasi et al., 2007). Root mean square of the error to standard deviation ratio with a value of less than 0.7 indicates satisfactory accuracy of a model (Ibarra‐Zavaleta et al., 2017).

The mean square prediction error was divided into MB and SB deviations to identify systematic biases as follows:

$$\text{MB}={\left(\bar{P}‐\bar{O}\right)}^{2}$$

$$\text{SB}={\left(S_{p}‐r\times S_{O}\right)}^{2}$$

where $\bar{P}$ is mean of predicted values, $\bar{O}$ is mean of observed values, *S*_p_ is the standard deviation of predicted values, *S*_o_ is the standard deviation of observed values and *r* is the coefficient of correlation. Low values of MB and SB indicate a small bias of the model.

The concordance correlation coefficient (CCC), a parameter of model accuracy, was calculated as follows:

$$\text{CCC}=r\times C_{b}$$

$$C_{b}={\left[\frac{\left(V+\frac{1}{V}+U^{2}\right)}{2}\right]}^{1}$$

$$V=\frac{S_{O}}{S_{P}}$$

$$U=\frac{\left(\bar{P}‐\bar{O}\right)}{\sqrt{S_{p}\times S_{O}}}$$

where $\bar{P}$ is the mean of predicted values, $\bar{O}$ is the mean of observed values, S*_p_* is the standard deviation of predicted values, S*_o_* is the standard deviation of observed values and *r* is the coefficient of correlation. The closer the CCC to 1, the more accurate the model (Niu et al., 2018). The CE was calculated for each constructed model as follows:

$$\text{CE}\%=100\times \left|\frac{O_{i}‐P_{i}}{O_{i}}\right|$$

where *O_i_* and *P_i_* are observed and predicted LWs, respectively. The validation set was used to calculate prediction error (PE) using the CE equation.

The frequency of negative residuals was presented to provide a better description of error distribution. The correlation between the residuals and LW was calculated.

Published models (Table 1) were assessed for accuracy using the data of this study. The LW of each sheep in our study was predicted using the corresponding model (considering breed and sex) and PE calculated. Data were analysed using R (R Core Team, 2017).

## 3 RESULTS

### 3.1 Model construction and validation

The range of LW was 16–60, 15–58 and 15–44 kg for Adilo, Bonga and Horro breeds, respectively. The range of HG was 52–90, 63–98 and 60–90 cm for Adilo, Bonga and Horro, respectively. The means (and standard deviations) of LW of Adilo, Bonga and Horro sheep were 30 kg (7.42 kg), 30.03 kg (6.09 kg) and 27.6 kg (6.06 kg), respectively. The means (and standard deviations) of HG Adilo, Bonga and Horro sheep were 71.7 cm (6.51 cm), 79 cm (5.84 cm) and 75.3 cm (6.17 cm), respectively.

**FIGURE 1:**
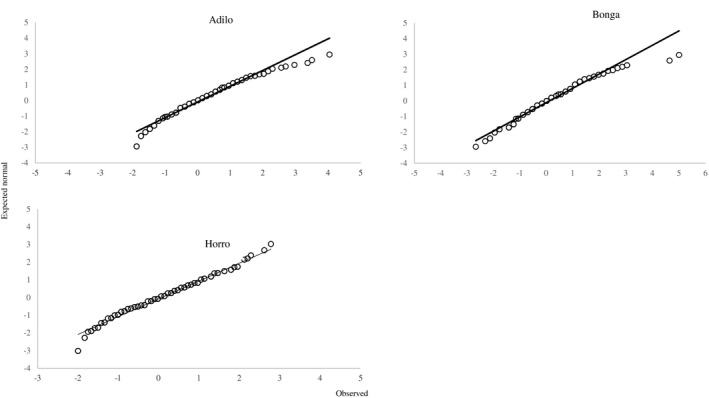
Q‐Q normal plot of live weight of Adilo, Bonga and Horro sheep

**FIGURE 2:**
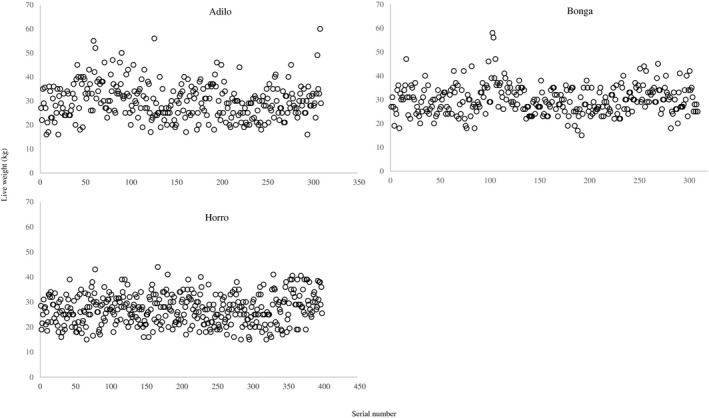
Live weight versus serial number of Adilo, Bonga and Horro breed

No outliers were found in the data. Visual inspection of normal probability plots showed that LW was normally distributed with some deviation in all sheep breeds (Figure 1). Figure 2 shows that there was no systematic relation between LW and the serial number of sheep in the three breeds. Puchwein algorithm identified 38 Adilo sheep, 39 Bonga sheep and 50 Horro sheep to be used as validation sets. Data of the remaining sheep were used to construct prediction models.

**FIGURE 3:**
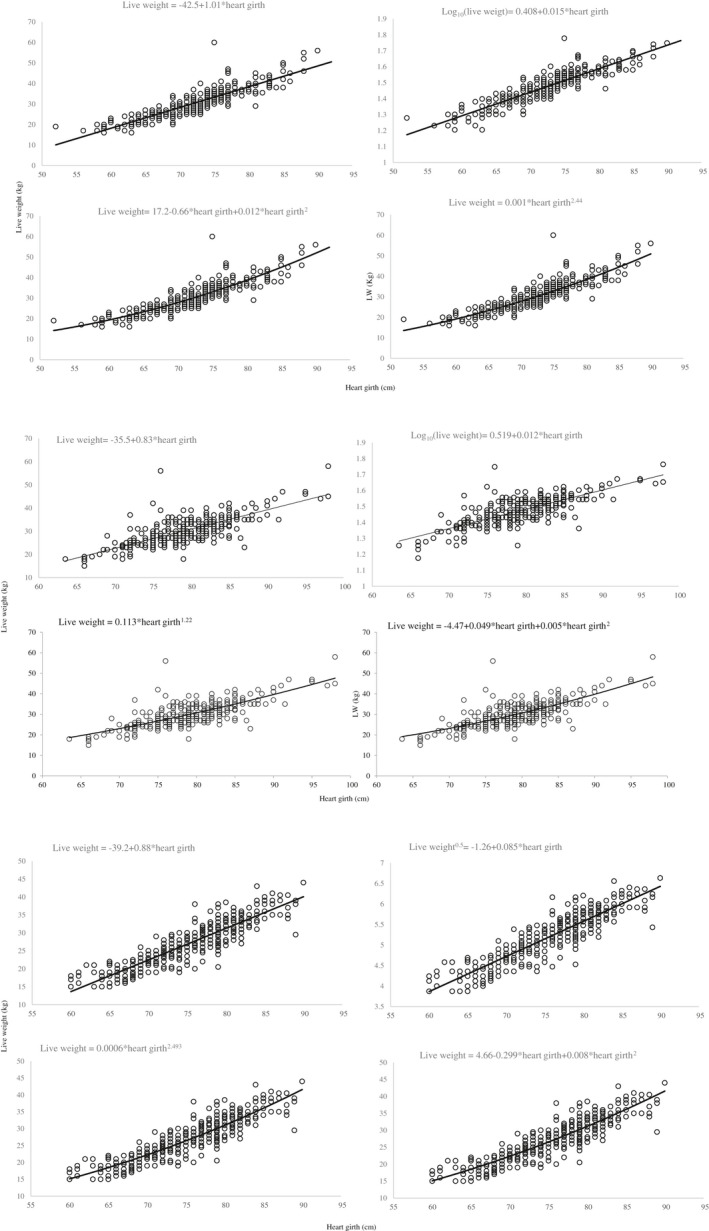
(a) Sheep live weights as a function of heart girth of Adilo breed. (b) Sheep live weights as a function of heart girth of Bonga breed. (c) Sheep live weights as a function of heart girth of Horro breed

Results of the Box‐Cox procedure showed that λ with the highest log‐likelihood values in all breeds was different from 1 (zero in Adilo and Bonga and 0.5 in Horro). Figure 3 presents selected models that depict the relationship between LW and HG in Adilo, Bonga and Horro sheep. Specifications and prediction performance parameters of constructed models are presented in Table 2 and Figure 3a–c, respectively. Constructed models of every breed had similar *R* ^2^ (0.79–0.82 in Adilo, 0.58– 0.59 in Bonga, 0.8–0.811 in Horro). Similar RSR values were found in all constructed models for the breeds (0.425 to 0.464 for Adilo, 0.632 to 0.661 for Bonga and 0.435 to 0.444 for Horro). All models of the breeds had low MB and SB (<0.001). All models of the breeds had high and similar CCC values ranging from 0.749 to 0.886 in Adilo, 0.723 to 0.734 in Bonga and 0.857 to 0.881 in Horro. Only Box‐Cox models of the three breeds had 95th percentile of CE ≤ 10.

**FIGURE 4:**
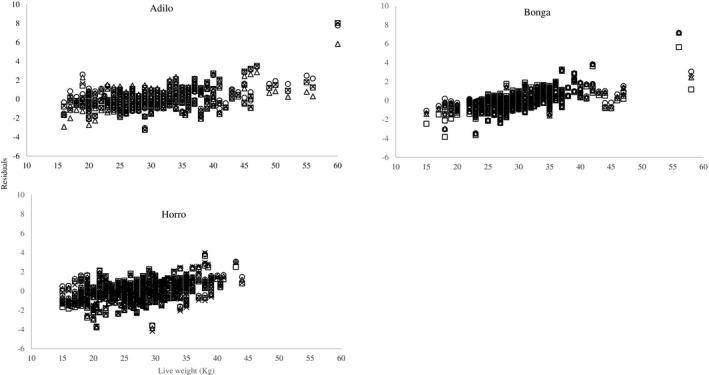
Standardized residuals versus live weight for the constructed models (o, simple linear model; □, transformed SLM; Δ, quadratic model; ×, allometric model) for individual breeds Adilo, Bonga and Horro

Results of the residual analysis are presented in Table 3. The relation between standardized residuals and LW was moderate and positive (*r* = 0.43–0.65; *p* <.001) for all constructed models of all breeds. Residuals of all constructed models were symmetrically distributed around zero (Table 3) without any drifts (Figure 4). In the three breeds, only simple linear models with Box‐Cox transformed LW had the 95th percentile of PE less than 10 (Table 3).

**TABLE 3:** Analysis of residuals of constructed and published models for Adilo, Bonga and Horro sheep

Breed	Models	Correlation with live weight	Negative residuals (%)	PE
Adilo	Linear	0.46	62	20.8
	Box cox (Log_10_)	0.43	51	6.3
	Allometric	0.45	57	33.6
	Quadratic	0.55	45	25.2
	*Published models*			
	Taye et al. (2016)	−0.53	39	21.2
Bonga	Linear model	0.65	55	23.7
	Box cox (Log_10_)	0.63	51	7.27
	Allometric	0.65	54	36.7
	Quadratic	0.65	55	42.2
	*Published models*			
	Edea et al. (2008)	0.54	48	53.9
Horro	Linear	0.442	49.5	21.3
	Box cox (LW^0.5^)	0.437	50	10
	Allometric	0.441	50.8	28.1
	Quadratic	0.483	50.3	23.6
	*Published models*			
	Edea et al. (2008)	−0.379	22.5	45.3

Note

All correlation coefficients were significant (*p* <0.05); PE: 95th percentile of prediction error.

### 3.2 Performance of published models

**FIGURE 5:**
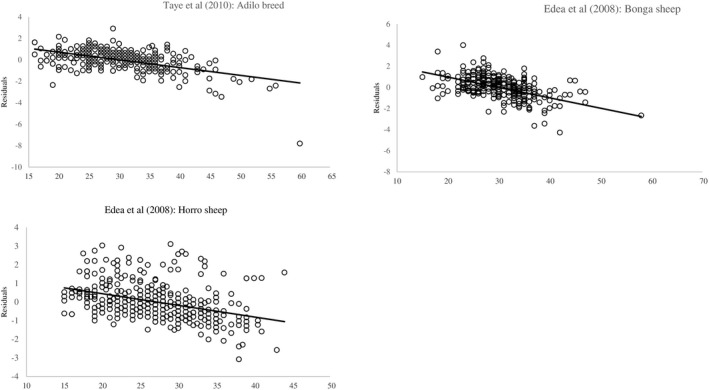
Standardized residuals versus live weight for the published models corresponding to Adilo, Bonga and Horro breed

Analysis of residuals of the published models are presented in Table 3 and Figure 5a–c. Models of (Edea et al., 2008) for Bonga and Horro breeds breed had a moderate correlation between residuals and LW.

In the Adilo breed, positive residuals were dominant in (Taye et al., 2016) model. In the Bonga breed, the number of positive residuals were almost equal to the number of negative residuals in the published model. In the Horro breed, positive residuals were dominant in (Edea et al., 2008) model. The PE of all percentiles was higher than 20 in published models for all breeds.

## 4 DISCUSSION

Calibrated weight bands based on models of published studies (Table 1) that are in use are perceived inaccurate by live animal market traders, particularly sheep traders. This has had several implications. First, traders have declared the bands redundant and resorted back to eyeball estimations. Consequently, inaccuracies of eyeball estimations have led to an ever‐growing mistrust of farmers towards market traders over selling prices of live animals. Farmers do not feel rewarded for their endeavours to fatten their sheep. This may pose a challenge to the current efforts to improve sheep fattening practices and adoption of sheep fattening technologies in the ongoing enhancement of the Sheep Value Chain Transformation being undertaken by the International Center for Agricultural Research in the Dry Areas (ICARDA) and its partners in Ethiopia.

The 95th percentile PE of the published models was higher than 20%. That means models of published studies had high margins of error in estimating LW of the three breeds of sheep which confirms observations of sheep traders in Ethiopia. As no parameters that describe the deviation of predicted LW from observed LW, such as PE of prediction models in the published studies was reported, it is difficult to explain the reason for their low performance. The distribution of LW in all breeds was normal with some deviation. That means a transformation of LW might be required to improve the predictability of simple linear models. That was confirmed by results of the Box‐Cox transformation procedure which showed that the best transformation of LW was base 10 logarithm for Adilo and Bonga and square root for Horro. Our results showed that all constructed models of the three breeds did not differ (*p* >0.5) in their *R* ^2^, RSR, MB, SB and CCC. However, these parameters are not enough to recommend the best fit model which predicts the LW of the three breeds with a satisfactory precision for veterinary and production applications. Therefore, residuals of constructed models were analysed for homogeneity and magnitude. Residuals in all constructed models were not related to the serial number of sheep. In other words, the accuracy of models was constant alongside the measurement process. The moderate correlation between residuals and LW in all constructed models suggests that the predictability of the models was independent of the LW of sheep in the 3 breeds.

Residuals correlated moderately and positively with LW (*p* <.05) which indicate that the heavier the animals, the higher the prediction error. However, CE and PE of the Box‐cox transformed models were lower than 10% for all breads. Therefore, this correlation has a negligible effect on the prediction accuracy of these models. Only CE and PE of all percentiles of Box‐Cox models was smaller than 10% which is the critical PE for purposes of veterinary services, management, breeding and nutrition (Goopy et al., 2017). Accordingly, Box‐Cox models could be used to get a robust estimation of LW in Adilo, Bonga and Horro sheep which would improve the bargaining power of Ethiopian farmers to get better prices for their sheep and may also increase the level of trust between farmers and animal traders in Ethiopia. The results of our study do not agree with (Goopy et al., 2017) who reported that the LW of cattle could not be predicted by HG with an appropriate accuracy to provide veterinary services and improve productivity. This contradiction may be a result of differences in morphology among livestock species in their relations between LW and HG.

Positive findings of this study generate support for further research to develop robust prediction models of LW based on HG or other body measurements for sheep breeds in developing countries.

## 5 CONCLUSIONS

Models produced in this study predicted LW of Adilo, Bonga and Horro sheep, using HG with PE less than 10% regardless of LW, age and sex. These models can be used to construct tables that contain HG corresponding to LW, thus enabling the production of calibrated weight bands of robust accuracy.

## ETHICAL STANDARDS

This study has been approved by the ethical committee of the International Centre of Agricultural Research in Dry Areas.

## CONFLICT OF INTEREST

The authors declare no conflict of interests.

## AUTHOR CONTRIBUTION

**Jane Wamatu:** Conceptualization; Data curation; Funding acquisition; Investigation; Methodology; Project administration; Resources; Supervision; Visualization; Writing‐original draft; Writing‐review & editing. **Ashraf Alkhtib:** Conceptualization; Data curation; Formal analysis; Investigation; Methodology; Project administration; Supervision; Visualization; Writing‐original draft; Writing‐review & editing.

## Data Availability

The datasets generated during and/or analysed during this study are available from the corresponding author on reasonable request.
